# Force Spectroscopy by Atomic Force Microscopy as Indicator for Cellular Microplastic Uptake

**DOI:** 10.3390/ijms27093770

**Published:** 2026-04-23

**Authors:** Tatjana Kolesnik, Kristin Öhlinger, Markus Absenger-Novak, Eleonore Fröhlich

**Affiliations:** Center for Medical Research, Medical University of Graz, 8010 Graz, Austria; tatjana.kolesnik@medunigraz.at (T.K.); kristin.oehlinger@medunigraz.at (K.Ö.); markus.absenger@medunigraz.at (M.A.-N.)

**Keywords:** spheroids, lung cancer, cell stiffness, polystyrene particles, dynamic exposure

## Abstract

Concentrations of microplastic particles (MPs) in the environment are low, and cellular uptake is difficult to measure. Cancer tissue accumulates more MPs than normal tissue. This study aims to determine whether cellular stiffness measurements by atomic force microscopy could indicate whether cells ingested MPs. In this study, spheroids with different compositions were exposed to MPs, and Young’s moduli were compared to fluorescent readings of MP uptake in monolayer cultures. The tested cancer cell lines differed in their basal Young’s modulus and in the increases observed upon MP exposure, both in monolayer and spheroid culture. Young’s moduli of the THP-1-containing spheroids were higher than those of spheroids without macrophages and were higher after MP exposure than before. In monolayer culture, softer cells showed larger increases in Young’s modulus after MP exposure than did stiffer cells. The Young’s moduli of the cell monolayers under static and dynamic conditions were positively correlated. Young’s modulus could serve as a parameter for MP uptake and can differentiate MP-containing spheroids from non-exposed spheroids. In monolayer culture, Young’s modulus can identify cell lines that ingested MPs. However, complicated use and low throughput limit the broad application of atomic force microscopy in biological evaluation.

## 1. Introduction

Microplastics are ubiquitous in the environment, and inhalation is thought to be the primary route of uptake of microplastic particles (MPs) compared with other exposure routes [1]. It is expected that MP exposure will increase because plastic production is still rising [2]. In general, indoor levels are higher than outdoor levels, but reported values are highly variable. One study reported 4.34 ± 1.93 plastic items (≤2.5 μm) per m^3^ in indoor rooms [3]. A review reported 1583 ± 1181 items per m^3^ without a size specification [4]. Particles with aerodynamic diameters between 1 and 5 μm are of particular importance for respiratory exposure because they can be deposited in the respiratory tract [5]. Compared with larger particles, they have a higher likelihood of being ingested by epithelial cells because epithelial cells have a low capacity for cellular uptake at sizes >1 μm [6]. Based on the assumption of moderate indoor activity (inhalation of 20 L of air) and a deposition rate of 30%, roughly 26 particles per day may be deposited in the lung. MP deposition may be relevant because a link between exposure to particulate matter 2.5 μm (PM_2.5_) and lung cancer has been reported [7]. Lung cancer tissues were found to contain more MPs than normal tissue [8]. Uptake and adverse effects of nano- and microplastic particles on A549 cell monolayers are relatively well studied [9], but few studies (e.g., [10]) evaluated MPs in 3D lung cultures. It is important to study interaction and uptake in 3D cultures because they are more representative of the in vivo situation.

We have generated well-characterized heterotypic spheroids consisting of A549, H1299, H23, H441, HCC827, and Calu-3 cells, MRC-5 fibroblasts, and the absence and presence of THP-1 macrophages [11]. In pilot experiments, we found that uptake of 1 μm fluorescently labeled polystyrene particles by the cancer cell/MRC-5 fibroblasts was irregular in static exposure. The number of particles ingested upon exposure in dynamic conditions was very low. Because 3D cultures are not suitable for analysis of fluorescence in whole mounts, we decided to use Young’s modulus to determine uptake. Uptake should result in an increase in Young’s modulus because plastics have a much higher Young’s modulus (e.g., polystyrene: 2.28–3.34 GPa; [12]) than cells. This method would also be useful to determine cellular uptake of environmental MPs, which are not fluorescent by themselves. Staining by Nile Red, 4-dimethylamino-4′-nitrostilbene (DANS), and Rose Bengal is suitable to stain MPs and to quantify them in environmental samples [13]. Similar methods can also be used to assess cellular effects of environmental MPs, but dye leaching and nonspecific staining of biological material are limitations. Further, these staining methods are not suitable for 3D cultures due to light scattering, quenching, signal overlap, and limited imaging resolution of 200 nm laterally and 500 nm axially [14]. In the previous study, we observed an increase in Young’s modulus in the cancer cell/MRC-5 spheroids after MP exposure and after the addition of THP-1 cells. However, it was unclear whether the increase was linked to MP uptake. To determine whether Young’s modulus could serve as an alternative indicator for uptake, spheroids of different compositions were assessed. Furthermore, Young’s moduli were compared with fluorescent evaluation in monolayer cultures.

In this study, exposure scenarios similar to those in the previous study on cancer cell/MRC-5 spheroids were used [11]. A concentration of 1 μg/mL was used because this concentration corresponds to the mean value of 1.07 μg polymer/mL determined in 68 blood samples [15]. Tilting rockers such as the IKA Rocker 2D tipping shaker at the settings used in this study produce only low shear stress in the range of 10^−3^ to 10^−1^ dyne/cm^2^, designed for gentle, low-shear mixing [16]. The study aimed to determine whether different lung cancer cells differ in MP uptake. The most common techniques for measuring cell stiffness (Young’s modulus) include the parallel-plate test and atomic force microscopy (AFM). Whereas the parallel-plate test is more suitable for analyzing a large sample, localized stiffness is better measured with AFM [17]. Young’s moduli were determined by AFM, which is an established technique for the planned application [18,19]. They were analyzed before and after MP exposure in the triple-cell spheroids in order to compare the data with those of the previously generated spheroids without THP-1. To validate Young’s modulus as a parameter for MP uptake, monolayers were used. Cell surface morphology of the cancer cells was studied, and the Young’s moduli of the MP-exposed cells were compared with fluorescent analysis of particle uptake.

## 2. Results

To determine whether the increase in Young’s modulus upon exposure to MPs could indicate uptake in spheroids, cancer cell/MRC-5/THP-1 spheroids were exposed to MPs in a manner similar to that in the pilot study with cancer cell/MRC-5 spheroids.

### 2.1. Composition of Spheroids

**Figure 1:**
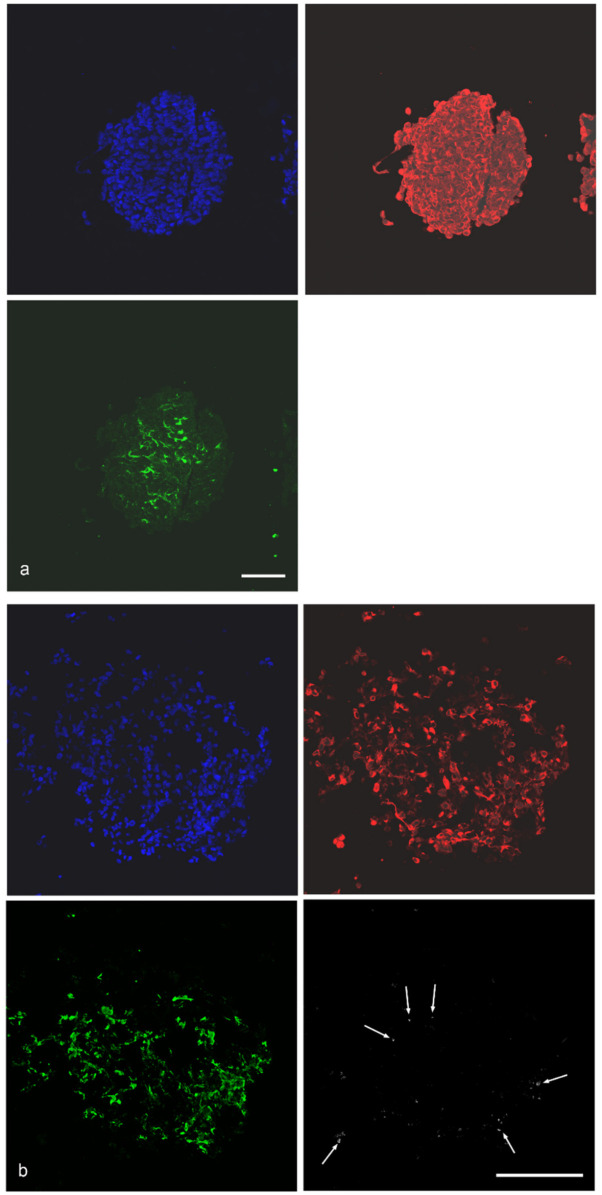
(**a**): Localization of cancer cells (anti-CK18 immunoreactivity, red) and fibroblasts (anti-fibronectin immunoreactivity, green) in H1299/MRC-5 spheroid. (**b**): Identification of cancer cells (anti-CK18 immunoreactivity, red) and fibroblasts (anti-fibronectin immunoreactivity, green) and THP-1 macrophages (anti-CD45 immunoreactivity, white) in H1299/MRC-5/THP-1 spheroids. The few anti-CD45-immunoreactive cells are indicated by arrows. Nuclei are counterstained with Hoechst 33342 (blue). Scale bar: 100 μm.

The distribution of cancer cells and fibroblasts was demonstrated in the pilot study [11]. Here, only spheroids containing H1299 cells are shown. Cancer cells are present throughout the spheroid, whereas MRC-5 fibroblasts are preferentially localized in the spheroid core (Figure 1a). THP-1 macrophages are predominantly localized at the spheroid periphery and may affect the interaction of the MPs with the spheroid (Figure 1b).

**Figure 2:**
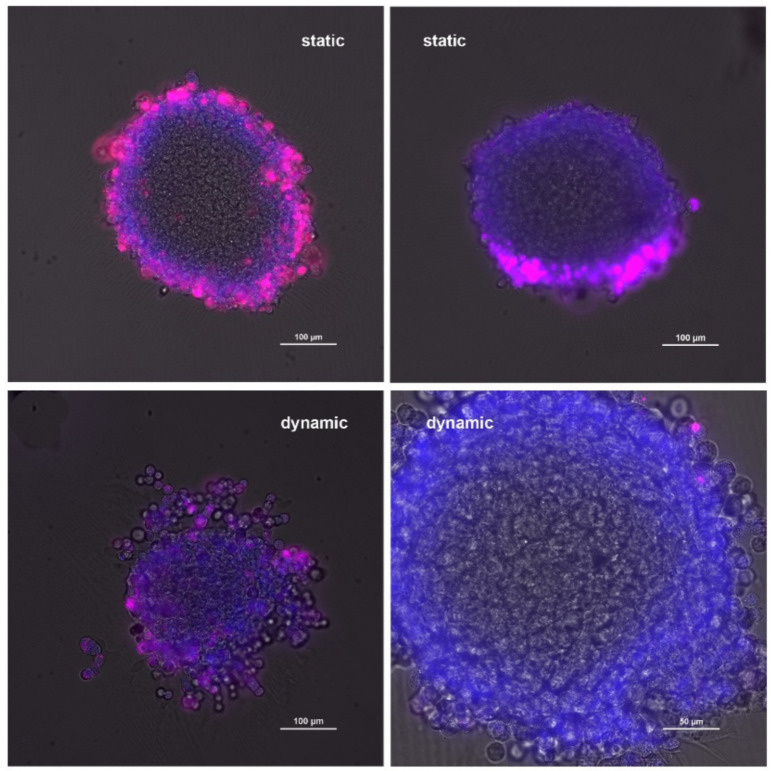
Localization of polystyrene particles (red) in H1299/MRC-5 spheroids after static and dynamic exposure. Nuclei are stained with Hoechst 33342 (blue). Scale bar: 100 μm except lower right image (50 μm).

To obtain qualitative information on MP uptake, vibratome sections of the spheroids were prepared. Vibratome sections are thicker than sections prepared after embedding in other media and enable assessment of a larger portion of the spheroid. When analyzing uptake in static exposure, it was found that uptake in some sections appeared homogeneous, whereas in others MPs were located at one pole of the spheroid (Figure 2). Furthermore, agglomerates were seen in all sections.

Upon dynamic exposure, minimal particle agglomeration was observed. Many sections did not contain any particles, while a few single particles were visible in some sections.

**Figure 3:**
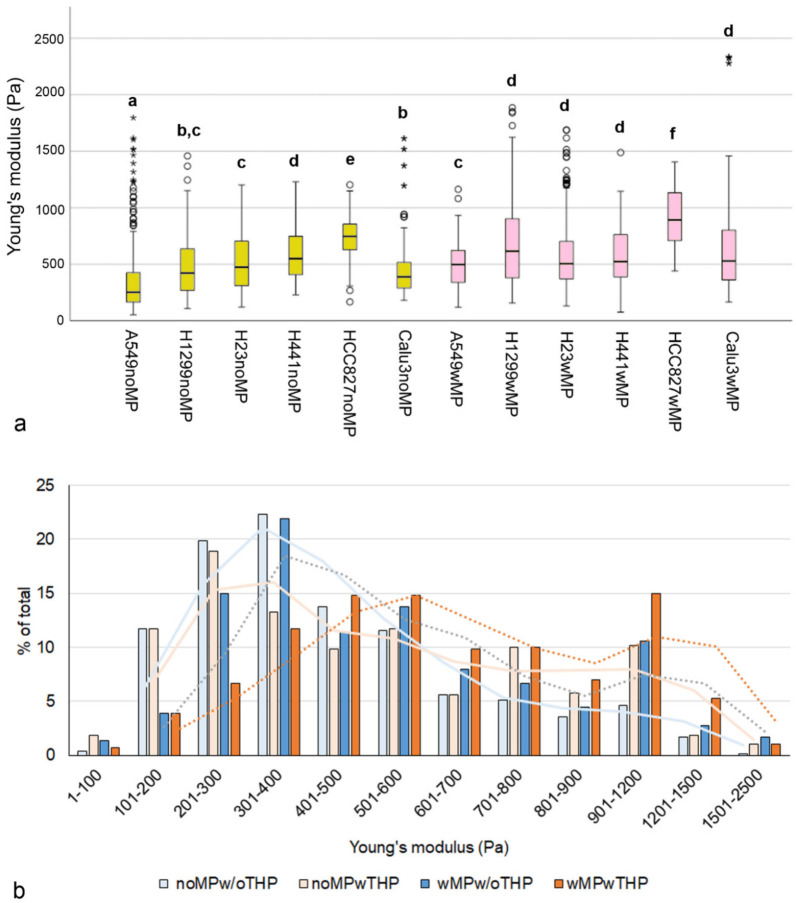
(**a**): Box plot showing the median, interquartile range (IQR), data range, and outliers in the Young’s modulus measurements. Circles represent values located between 1.5× and 3× the IQR above Q3 or below Q1. Asterisks represent values that are more than three times the IQR above Q3 or below Q1. Groups with significant differences (*p* < 0.05) are designated by letters. Cancer cell/MRC-5/THP-1 spheroids not exposed (noMP) and exposed to polystyrene particles (wMP) are shown. (**b**): Classes of Young’s moduli in cancer cell/MRC-5 spheroids and cancer cell/MRC-5/THP-1 spheroids without MP exposure (noMPw/oTHP and noMPwTHP) and both types of spheroids with MP exposure (wMPw/oTHP and wMPwTHP). The analysis included hundred measurements for each spheroid type and condition. Curves show moving averages in the corresponding colors: cancer cell/MRC-5 spheroids as solid lines and cancer cell/MRC-5/THP-1 spheroids as dotted lines.

Significantly higher Young’s moduli (*p* < 0.001) were observed in MP-exposed than in non-exposed spheroids for all spheroid types, except those containing H441 (Figure 3a).

To evaluate Young’s modulus as a potential indicator of MP uptake, 100 values for each cell line in each culture and exposure condition were randomly selected. The distribution of Young’s moduli shifted to higher values upon MP exposure (Figure 3b). The median ± median absolute deviation (MAD) was 385 ± 131 Pa for noMPw/oTHP spheroids, 462 ± 215 Pa for noMPwTHP, 472 ± 171 Pa for wMPw/oTHP, and 591 ± 202 Pa for wMPwTHP spheroids. Differences between MP-exposed and non-exposed spheroids were statistically significant (*p* < 0.001). The increase in Young’s modulus in THP-1-containing spheroids was greater (119 vs. 87 Pa) than in spheroids without THP-1 macrophages.

### 2.2. Characterization of Cancer Cell Monolayers

The higher particle uptake by cells grown on soft substrates [20] suggests that cell stiffness could affect MP uptake. Further, cell surface morphology may be relevant. Therefore, the cell lines used were characterized with respect to surface morphology.

**Figure 4:**
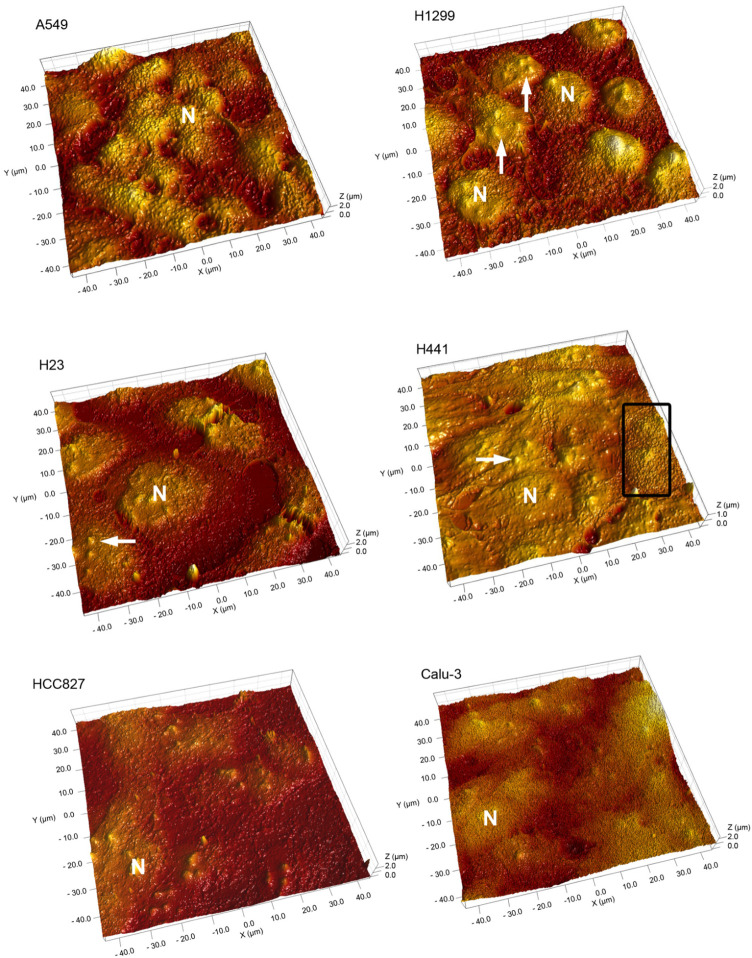
3D image of lung cell surfaces cultured in monolayers. Small protrusions are visible, and nuclei (N) containing nucleoli (arrowheads) can be identified. Small areas in the H441 monolayer show dense microvilli (rectangle). Area 40 × 40 μm, height 2 μm, except H441 cells.

Figure 4 shows the surface of the cell monolayers, where nuclei with nucleoli and surface protrusions can be discerned. The height of the surface protrusions is estimated at around 0.5 μm, suggesting they represent microvilli. Microvilli are particularly expressed on mucus-producing cells of the respiratory tract and are also formed by cancer cells [21,22], where their density correlates with malignancy. Whereas the coverage with microvilli was homogenous in the monolayers of most cell lines, marked intercellular differences were evident in the H441 monolayers (Figure 4).

### 2.3. Determination of MP Uptake in Monolayers

#### 2.3.1. Fluorescence Microscopy

**Figure 5:**
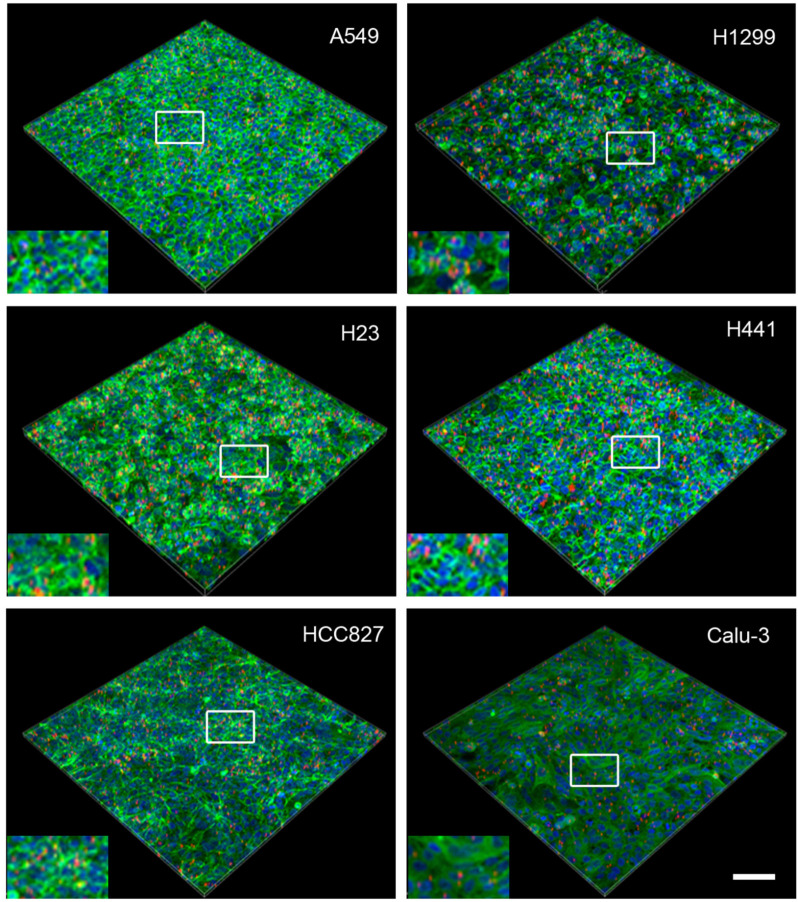
Images of 3D projections of cell monolayers exposed to polystyrene microparticles (red) in static exposure. The cells are labeled with Alexa 488 phalloidin (green), and the nuclei are counterstained with Hoechst 33342 (blue). Scale bar: 100 μm. The white boxes indicate the location of the inset.

**Figure 6:**
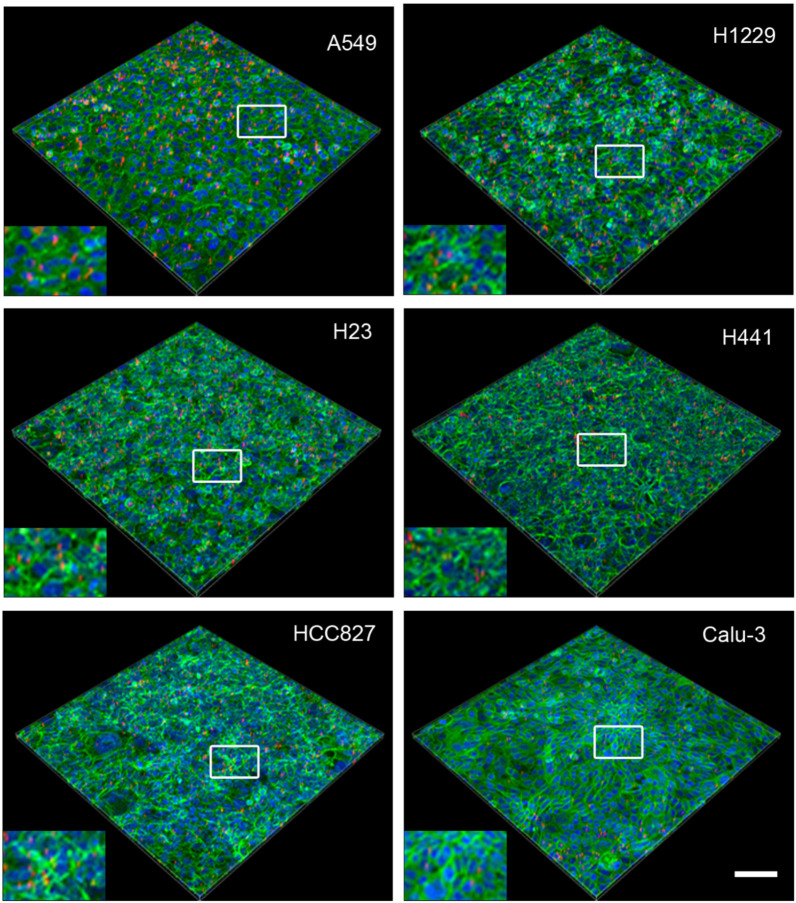
Images of 3D projections of cell monolayers exposed to polystyrene microparticles (red) in dynamic exposure. The cells are labeled with Alexa 488 phalloidin (green), and the nuclei are counterstained with Hoechst 33342 (blue). Scale bar: 100 μm. The white boxes indicate the location of the inset.

To validate Young’s modulus for determining MP uptake, semi-quantitative analysis using confocal scanning microscopy was performed. The uptake was compared between static and dynamic conditions. The application of shear stress to cells by fluid movement can alter cell physiology [23]. Shear stress during dynamic exposure in the tipping shaker is considerably lower (0.81 dyne/cm^2^) than that applied in the simulation of capillary flow [24]. Nevertheless, MP exposure was restricted to 24 h to prevent the changes in morphology, motility, proliferation, apoptosis, and protein expression that were induced by linear shear stress of 4–12 dyne/cm^2^ for 40 h in confluent endothelial monolayers. Microscopic images show that static uptake was significantly higher in most cell lines (Figure 5 and Figure 6).

**Figure 7:**
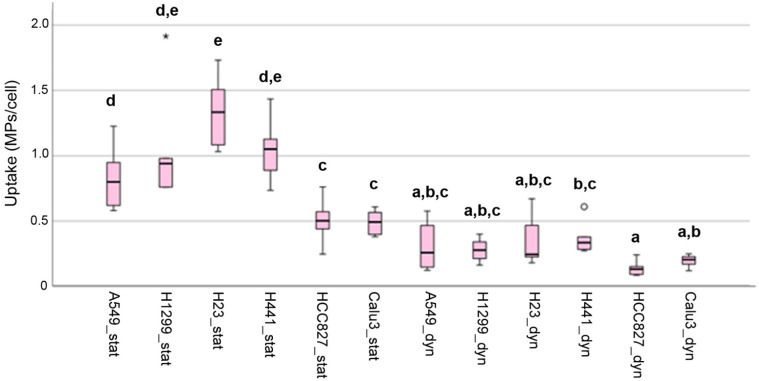
Box plot showing the median, interquartile range (IQR), data range, and outliers in the cellular uptake of MPs according to analysis by confocal microscopy. Circles represent values located between 1.5× and 3× the IQR above Q3 or below Q1. Asterisks represent values that are more than three times the IQR above Q3 or below Q1. Cells in static exposure (suffix “stat”) and dynamic exposure (suffix “dyn”) are shown. Letters indicate groups that are significantly different (*p* < 0.05).

The semi-quantitative analysis of the images showed that HCC827 and Calu-3 cells ingested significantly fewer MPs than A549, H1299, H23, and H441 cells in static exposure (Figure 7). There were no significant differences between the cell lines in MP uptake under dynamic exposure. In general, uptake upon static exposure was 2–5 times higher than in dynamic exposure (Figure S1, Supplementary Materials).

#### 2.3.2. Cell Stiffness (Young’s Modulus)

**Figure 8:**
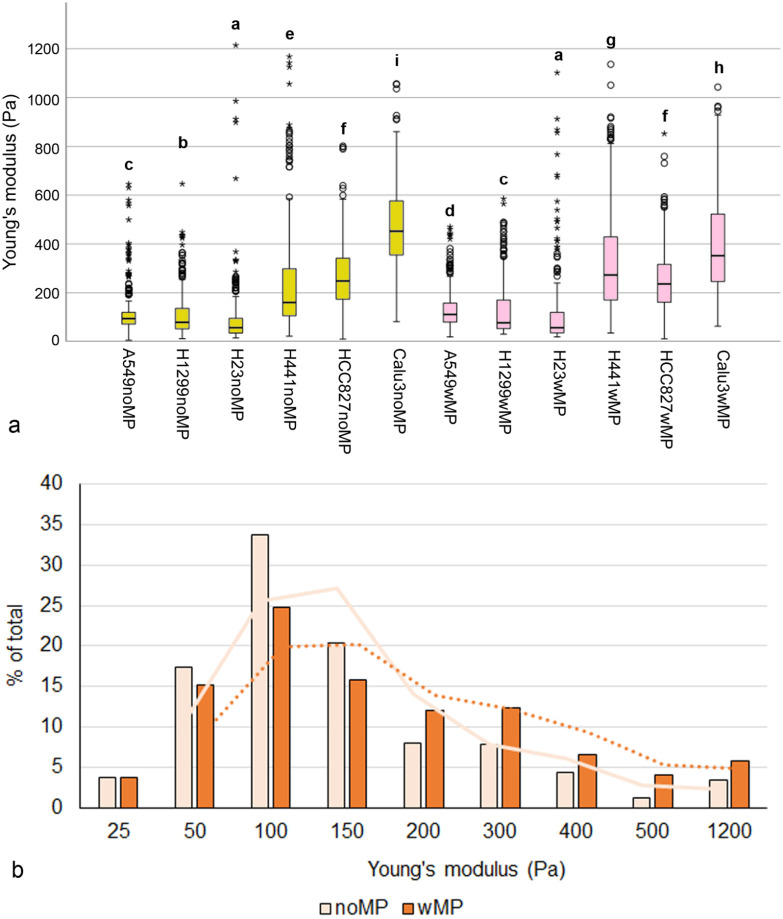
(**a**): Box plot showing the median, interquartile range (IQR), data range, and outliers in the Young’s modulus without (suffix “noMP”) and after dynamic exposure (suffix “wMP”) of the cell in a monolayer to MPs. Circles represent values located between 1.5× and 3× the IQR above Q3 or below Q1. Asterisks represent values that are more than three times the IQR above Q3 or below Q1. Letters indicate groups that are significantly different (*p* < 0.05). (**b**): Distribution of Young’s moduli in A549, H1299, H23, and H441 monolayers exposed (wMP) and not exposed (noMP) to particles. Curves represent moving averages in the corresponding colors: non-exposed cells are solid lines, and MP-exposed cells are dotted lines.

Cell stiffness shows regional variation. Nuclear regions are generally softer than peripheral regions [25] due to cytoskeletal heterogeneity. Further, differences have also been reported between cellular regions in the composition of the glycocalyx [26]. Because both regions were measured in the monolayers, the Young’s modulus shows significant variation (Figure 8a). By choosing very soft cantilevers (0.01 N/m), mainly the outer coat of the cells—the glycocalyx, which is the layer that first contacts the particles—was probed. Figure 8a shows that A549, H1299, and H23 were significantly softer than H441, HCC827, and Calu-3 cells.

To address whether Young’s modulus could serve as an indicator for MP uptake, Young’s moduli of the cell lines showing the highest uptake rates in static exposure by microscopic detection (e.g., A549, H1299, H23, and H441) were used. The medians of Young’s moduli with and without MP exposure were significantly different (*p* < 0.001), and the distribution of MP-exposed cells is shifted to higher Young’s moduli (Figure 8b).

Young’s moduli were determined only after dynamic exposure to MPs, when it is expected that MPs had been ingested. Cell stiffness compared to in non-exposed cells was significantly increased in A549, H1299, and H441 cells (Figure 8b).

### 2.4. Correlation Between MP Uptake and Cellular Parameters in Monolayers

Correlation between Young’s moduli with and without MP exposure was significant (*p* < 0.001), but not strongly monotonically correlated (ρ < 0.51). Further, Young’s moduli of MP-exposed cells in dynamic exposure were correlated to static uptake in fluorescence analysis (*p* < 0.004). By contrast, Young’s moduli of non-MP-exposed cells were strongly negatively correlated (ρ = −0.802) to particle uptake in static exposure (*p* < 0.001).

## 3. Discussion

The results of this study show significant differences in MP uptake between cancer cells in both monolayers and heterotypic spheroids. While there is a general correlation between uptake in monolayers and spheroids, some cancer cells, such as H441 cells, changed their uptake from high in monoculture to low in 3D culture. Among the six cell lines tested, uptake in H441 cells was relatively high under both static and dynamic exposure but low in spheroid cultures.

Young’s modulus could serve both as a predictor and as a parameter for qualitative evaluation of MP uptake. Different techniques are used to determine particle uptake by cells, with confocal microscopy and flow cytometry being the most commonly used [27]. We preferred confocal microscopy to flow cytometry to semi-quantify MP uptake to prevent quenching at higher intracellular particle concentrations. Both methods present the risk of measuring particles that are attached rather than ingested [28]. Occasional attached particles were seen with AFM in static exposure, but not in dynamic exposure. Since this error is linked to the methodology, it should not bias the intercellular comparison.

### 3.1. Young’s Modulus as Predictor of MP Uptake in Monolayers

The negative correlation between Young’s modulus and MP uptake in our study suggests that softer cells ingest more particles. When grown on the same substrate, Young’s moduli of more malignant breast cancer cells were found to be softer than those of less malignant cells [29]. The measurement conditions used in this study (e.g., tip radius, spring constant of 0.2 N/m and indentation of ≤200 nm) enable the assessment of the glycocalyx, plasma membrane, and the actin cortex [30]. The glycocalyx, the sugar-rich coating of the outer surface of the plasma membrane of tumor cells, differs in composition between tumor cells, and MUC1 surface expression is correlated with increased invasiveness in breast cancer cells [31]. More aggressive cancer cells have a thicker glycocalyx than less malignant cells [32]. This would indicate that softer cells with a thicker glycocalyx ingest more MPs than cells with a thinner glycocalyx. We did not investigate whether H23 cells showing the highest MP uptake are more aggressive than the other cells. However, it has been reported that H23 cells secrete more IL-8 than the H552 lung cancer cell line [33]. The higher basal levels of IL-8, an autocrine and angiogenic growth factor, may indicate a higher malignant potential than in other lung cancer cell lines. This finding would correspond to the report of a higher content of MPs in lung cancer than in normal lung tissue [8]. The relevance of the glycocalyx for plastic particle uptake in endothelial cells was demonstrated differently: in these cells, enzymatic removal of the glycocalyx increased the uptake of 50 nm polystyrene particles [34].

### 3.2. Young’s Modulus as Indicator of MP Uptake in Spheroids

The comparison of Young’s moduli before and after MP exposure showed that the mean Young’s moduli of MP-exposed spheroids were significantly higher than those of spheroids without MP exposure. The increase in THP-1-containing spheroids was greater than in cancer cell/MRC-5 spheroids. A potential explanation is that phagocytes ingest 1 μm particles faster than cancer cells, such as A549 cells [35]. This confirms the hypothesis that MP intake caused the increase in Young’s modulus and that the determination of Young’s modulus could identify MP uptake.

For validation, Young’s moduli of cells showing significant increases in the number of fluorescent MPs in static conditions were compared to those of their non-exposed counterparts. Static exposures were not analyzed because adhering, non-ingested, MPs complicated the measurements (e.g., extreme increases in Young’s moduli and loss of contact between cantilever and cell). The final assessment of the use of Young’s modulus for MP uptake is not possible because cell lines showed significant differences in the microscopic assessment only upon static and not upon dynamic exposure. It was also noted that the increase in static compared to dynamic exposure differed between the cell lines. Decreased membrane tension and increased glycocalyx thickness, causing cell softness, often co-exist in cancer cells [32,36]. However, both parameters are not strictly linked and may explain the nonlinear relationship between MP uptake in static and dynamic conditions.

**Figure 9:**
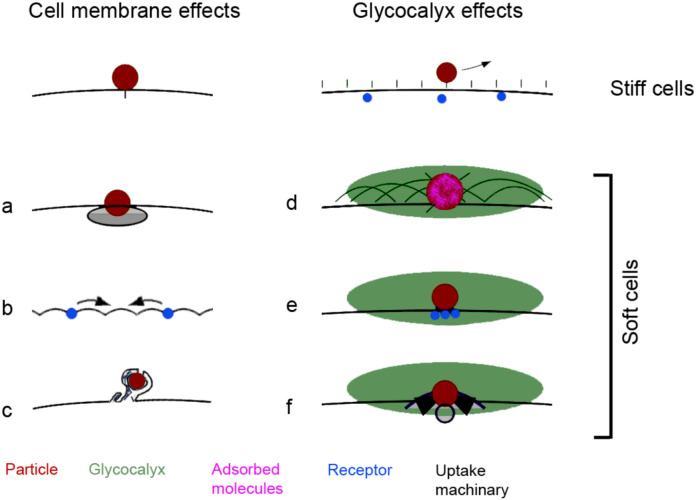
Mechanisms that contribute to the higher particle uptake by soft cells compared to stiffer cells. (**a**): Larger contact area; (**b**): higher receptor mobility; (**c**): higher membrane ruffling; (**d**): decreased diffusion and opsonization by macromolecule adhesion; (**e**): higher receptor clustering; (**f**): upregulated uptake machinery. The arrow indicates that particles can detach from the cell.

Several mechanisms may explain why softer cells show higher MP uptake (Figure 9). These mechanisms include easier deformation, resulting in a larger contact area with the particles, thereby prolonging cell–particle contact; increased receptor mobility; higher large-scale uptake (e.g., micropinocytosis) by facilitating membrane ruffle and cup formation; and hindrance of particle diffusion [32,37,38].

### 3.3. Role of MP Exposure

MP uptake was higher in the static condition than in the dynamic exposure. This finding is consistent with the expectation that cellular uptake requires attachment, which is presumably stronger in static conditions because contact is facilitated by sedimentation. Uptake of positively and negatively charged silica particles (350 nm) at high exposure doses of 100–200 μg/mL was approximately 1.5 times lower in dynamic conditions (tipping shaker) than in static conditions [39]. However, the situation may be different when larger agglomerates are formed that are not easily taken up by cells. In that case, flow conditions may facilitate dispersion and lead to higher cellular uptake rates [40].

In dynamic conditions, particle contact is short, and it is likely that plasma membrane properties—such as surface charge, glycocalyx structure, and receptor distribution (e.g., epidermal growth factor receptor)—determine interaction with MPs. Furthermore, different compositions/charges of the glycocalyx may play a role in the binding of MPs. For example, MUC1, which is more highly expressed in more malignant cancer cells, due to its high amounts of sialic acid residues, is characterized by a strong negative charge [41].

### 3.4. Settings for Spectroscopy of Spheroids and Monolayers

Spectroscopic AFM methods were developed for the measurement of hard, dry, homogeneous materials. Biological samples are soft, hydrated, heterogeneous, and dynamic, making the spectroscopic response weaker, more complex, and more sensitive to perturbation [42]. In summary, limitations include problems with the instrument, such as slow speed, tip convolution, artifacts, mechanical and electrical noise, and complex calibration [43]. In biological materials, mechanical damage to the cells, immobilization of tissues, environmental instability, and tip contamination are challenging [44]. Immobilization methods (e.g., various coatings and gels) proved not to be efficient enough to immobilize the spheroid for the duration of the measurements. However, immobilization by placing spheroids in a 250 μm micromesh array was effective. This immobilization method posed a limitation for cantilevers with specific properties. Custom tipless cantilevers, for instance, in the required length of 450 μm to reach the top of the spheroid, were only available without coating. Reflective coatings on cantilevers are necessary for our AFM because it is designed to work with an optical beam-deflection system. This region was analyzed because other areas were not accessible due to the curvature; therefore, deeper layers of the spheroids could not be probed. For spectroscopy of the monolayers, softer cantilevers with a small spherical tip (1.98 μm) were used to avoid damaging the cells.

## 4. Materials and Methods

MRC-5 (ATCC-CCL-171; LGC Standards GmbH, Wesel, Germany) cells were used in passages 27–38, THP-1 cells (ATCC-TIB-202; Cell Line Services, Eppelheim, Germany) in passages 13–23, A549 (ATCC-CCL-185; Deutsche Sammlung für Mikroorganismen und Zellkulturen GmbH, Braunschweig, Germany) cells in passages 24–67, H1299 (ATCC-CRL-5803; LGC, Luckenwalde, Germany) cells in passages 62–81, H23 (ATCC-CRL-5800; LGC) in passages 103–121, H441 (ATCC-HTB-174; LGC) cells in passages 7–18, HCC827 (ATCC-CRL-2868; LGC) cells in passages 13–28, and Calu-3 (ATCC-HTB-55; LGC) cells in passages 19–24. A549 cells were cultured in the DMEM; H1299, H23, HCC827 and THP-1 in RPMI 1640; and Calu-3 and MRC-5 in MEM + 1% L-glutamine and RPMI 1640. All media were supplemented with 10% FBS and 1% penicillin/streptomycin.

### 4.1. Generation of Spheroids

#### 4.1.1. Lung Cancer Cells/Fibroblasts

For lung cancer cell/fibroblast co-culture, lung cancer cells and fibroblasts were seeded with 2500 lung cancer cells and 5000 fibroblasts in DMEM + 10% FBS into Nunclon Sphera 96F-bottom ultralow attachment (ULA) plates (Thermo Fisher Scientific, Vienna, Austria) and cultured for 72 h without medium changes.

#### 4.1.2. Lung Cancer Cells/Fibroblasts/Macrophages

For triple cultures, THP-1 monocytes were differentiated into M0 macrophages by treatment with 10 nM phorbol 12 myristate (PMA) in RPMI 1640 for 48 h. Nonadherent cells were removed by medium exchange, and cells were post-incubated for 24 h. Polarization to M2 macrophages was achieved by treatment with a cocktail of 20 ng/mL IL-4 (Eubio, Vienna, Austria) and 20 ng/mL IL-13 (Eubio, Vienna, Austria) for 48 h. Cells were harvested by washing with 0.05% trypsin EDTA and resuspended in PBS + 2% FBS. Cells were incubated for 30 min at 4 °C with PE-CD209 (BD Biosciences, Vienna, Austria, 1:20), washed with PBS + 2% FBS, and sorted on a BD FACSAria Ilu (BD Biosciences, Vienna, Austria). Cancer cells and MRC-5 fibroblasts were seeded at a 1:2 ratio (2500 cancer cells + 5000 fibroblasts) and cultured in DMEM + 10% FBS. After 24 h, 2500 M2 cells were added, and cultures were continued for an additional 48 h. Cancer cell/MRC-5 spheroids measured 350–450 nm, and cancer cell/MRC-5/THP-1 spheroids measured 520–650 nm [11]. Immunocytochemical staining protocols are described in the Supplementary Materials.

### 4.2. Atomic Force Microscopy

#### 4.2.1. Spectroscopic Measurements of Spheroids

Spheroids were transferred from the cultivation plate into a fresh 96-well ULA plate and washed three times with medium without FBS. After washing, each spheroid was transferred to a well of a micromesh array (well size: 250 μm; Micro Surfaces GmbH, Kornwestheim, Germany) containing 100 μL CO_2_-independent medium (Gibco, Grand Island, NY, USA, 18045-088) without supplements. The micromesh array was placed in a 50/40 WillCo dish (WillCo Wells B.V., Amsterdam, The Netherlands) and 3 mL of CO_2_-independent medium was added. The assembly was placed on an inverted Axio Observer Z1 microscope (Zeiss, Jena, Germany) for AFM using a Nanosurf Flex-Bio system (Nanosurf, Liestal, Switzerland). A Contact cantilever Cont-GD-G (Budget Sensors, Sofia, Bulgaria) with a resonant frequency of 13 kHz, tip radius of 10 nm, and a nominal spring constant of 0.2 N/m was used. A new cantilever was calibrated before each experiment to obtain the spring constant using the software-integrated Sader method [45]. In liquid, the deflection sensitivity of each cantilever was measured on the cover glass as background and calculated in the Nanosurf C3000 v3.10.0.23 software. Force spectroscopy was performed with a ramp size of 50 μm, an approach/retract speed of 5 μm/s, and a maximum force of 20 nN. For determination of Young’s modulus, 100–200 individual curves from A549, H1299, H23, H441, HCC827, and Calu-3 cells were analyzed. Bias from contamination of the cantilever with MPs was prevented by microscopic control of the tip after each spheroid. The tip dimensions were not checked before and after the measurements because we did not use high loads, abrasive samples, or chemical environments. Spheroids were analyzed using AtomicJ v1.4.1 (Hermanowicz et al.) [46]. A Poisson’s ratio of 0.5 for mammalian cells was used [47], and Young’s modulus based on the Sneddon model for conical/pyramidal indenters [48] was calculated as

$$E=\frac{\pi \cdot \left(1\backslash -v^{2}\right)\cdot F}{2\cdot \tan\left(\alpha \right)\cdot \delta^{2}}$$

E = Young’s modulus, F = applied force (N), δ = indentation depth (m), ν = Poisson’s ratio of the cells (0.5), and α = half-opening angle of the conical tip.

#### 4.2.2. Spectroscopic Measurements of Monolayers

Confluent cell layers in TPP^®^ Petri dishes (Sigma-Aldrich GmbH, Vienna, Austria) were analyzed in CO_2_-independent medium without supplements using a Nanosurf FlexBio system (Nanosurf, Liestal, Switzerland). Cantilever CP-qpSCont-PS-A with an 11 kHz resonant frequency, and a nominal spring constant of 0.01 N/m (NanoAndMore GmbH, Wetzlar, Germany) was used. Prior to the measurements, the cantilevers were freshly coated with 0.1% Pluronic F127 in aqua dest. for 10 min (Sigma-Aldrich) to reduce adhesion to the plasma membrane. For each measurement, the cantilever was replaced; the spring constant and deflection sensitivity were determined at each cell monolayer. Spectroscopy was performed with a ramp size of 4 μm, a speed of 1 μm/s, and a stop value at 300 pN. The average indentation depth was up to 200 nm. In total, 449–629 individual curves of untreated and 300–700 curves of MP-treated A549, H1299, H23, H441, HCC821, and Calu-3 cells were analyzed. Bias from contamination of the cantilever was prevented by microscopic control of the tip after each monolayer. The force curves were calculated and analyzed using the SPIP analysis software v6.6.4 (Image Metrology, Horsholm, Denmark) and reported as Young’s modulus.

Young’s modulus was calculated as for spheroid measurements.

#### 4.2.3. Imaging of Monolayers

Confluent cell layers in TPP^®^ Petri dishes were briefly washed twice with PBS, then fixed with 4% formalin. The imaging protocol was performed according to Francis et al. [49]. After a short rinse in PBS, they were rinsed in aqua dest. to avoid crystal formation, and then in 70%, 90%, and 100% EtOH. After a short treatment with hexamethyldisilazane, sections were air-dried under a chemical hood. The cell monolayers were imaged in the Nanosurf ANA Box in tapping mode using the cantilever Tap300Al-G with a resonant frequency of 300 kHz and a nominal spring constant of 40 N/m (NanoAndMore GmbH). Image size was up to 90 μm, the setpoint was selected with 60–75% and vibration-free amplitude was about 1.98 V. The images were processed for a 3D presentation in SPIP software.

### 4.3. Incubation with Polystyrene Particles

#### 4.3.1. Hydrodynamic Size and Zeta Potential

FluoSpheres™ Polystyrene Microspheres, 1.0 μm red fluorescent (ex580/em605) from Thermo Fisher Scientific were used. Hydrodynamic size and zeta potential were determined by Photon Correlation Spectroscopy and Laser Doppler Velocimetry using a ZetaSizer Nano-ZS (Malvern Instruments, Malvern, UK) as reported previously [50]. Hydrodynamic size was measured with a 532 nm laser and a detection angle of 173°, and the dynamic fluctuations in light-scattering intensity caused by Brownian motion of the particles were evaluated. The zeta potential was calculated from the electrophoretic mobility by applying the Henry equation. Particle size in DMEM, in which they were applied to the cells, was 1271 nm, PDI was 0.453, and zeta potential was −19.2 mV. Solutions were freshly prepared and treated for 20 min in an ultrasound water bath (Elmasonic S40; ultrasonic frequency: 37 kHz, 40 W; Elma, Singen, Germany) before the measurements and exposures.

#### 4.3.2. Exposure of Spheroids to Particles

Spheroids were exposed to the FluoSpheres™ under the following conditions: one spheroid per well of a 12-well plate and treatment in 1 mL of a 1 μg/mL particle suspension in DMEM under static exposure or agitated on an IKA Rocker 2D tipping shaker (IKA Werke GmbH, Staufen im Breisgau, Germany) at 35 rpm for 24 h at 37 °C. After exposure, spheroids were fixed for 60–90 min with 4% paraformaldehyde. After three washes with PBS, spheroids were permeabilized with 0.1% Triton X-100 in PBS, washed three times in PBS, and subsequently incubated with Hoechst 33342 (1 μg/mL) for 30 min at RT.

The spheroids were suspended in 100 μL of 10% porcine skin gelatin in PBS (Sigma-Aldrich, Vienna, Austria), and the spheroid suspension was transferred to a Metal Base mold (Sakura Finetek Europe, Alphen aan den Rijn, The Netherlands). Additional embedding medium was added to a total volume of 400 μL. After cooling to RT (~30–60 min), the spheroids were ready for cutting using a vibratome VT 1200S (Leica Biosystems, Nussloch, Germany). Sections (40–80 μm) were prepared with commercially available razor blades, blade frequency of 85 ± 10 Hz, blade amplitude of 1–1.5 mm, and advance speed of 0.10–0.4 mm/s. Sections were harvested in PBS.

Images were taken at a Nikon A1R confocal microscope (Nikon CEE GmbH, Vienna, Austria) with Plan Apo λ 20× objective for overview and Apo 60× Oil λS DIC N2 for analysis. Ex/em of 409 nm/450 nm was used for Hoechst 33342 and 562 nm/592 nm for the Fluorospheres™. Image acquisition was performed with Nikon NIS-Elements software (version 5.22.22), and analysis was performed using the JOBS module.

#### 4.3.3. Exposure of Monolayers to Particles

Confluent monolayers were generated by seeding of 7.5 × 10^5^ A549 cells, 7 × 10^5^ H1299 cells, 1.2 × 10^6^ H23 cells, 3 × 10^6^ H441 cells, 1.6 × 10^6^ HCC827 cells, and 3.2 × 10^6^ Calu-3 cells in 24-well imaging plates (microscopic analysis) and TPP^®^ culture dishes (AFM). After 24 h, when cell layers were confluent, 1 μg/mL Fluospheres™ in 1 mL DMEM were added for 24 h at 37 °C either under static conditions or with agitation on an IKA Rocker 2D tipping shaker (IKA Werke GmbH). The IKA Rocker 2D performs a gentle back-and-forth rocking motion with an adjustable tilt angle and speed. Experimental data have been generated by Zhou et al. and were adapted to our condition [51]. Based on fluid height (5.2 mm), a 7° tilt angle, and 35 rpm (0.58 cycles/s), the shear stress was calculated to be 0.81 dyne/cm^2^. After the incubation, the cells were fixed for 15 min with 4% paraformaldehyde. After three washes with PBS, the cells were permeabilized with 0.1% Triton X-100 in PBS, washed three times in PBS again, and subsequently incubated with Alexa Fluor 488 Phalloidin (Thermo Fisher Scientific, 1:100) in antibody diluent and Hoechst 33342 (1 μg/mL) for 30 min at RT. Images were taken at a Nikon A1R confocal microscope (Nikon CEE GmbH) with Plan Apo λ 20× objective and ex/em of 409 nm/450 nm for Hoechst 33342, 488 nm/525 nm for Alexa Fluor 488, and 562 nm/592 nm for the Fluorospheres™.

For analysis, maximum intensity projections (MIPs) were generated from z-stacks with a thickness of 12–20 μm. Predefined segmentation parameters were applied to identify nuclei and MPs. Image processing and object counting were performed using NIS-Elements Nikon Analysis/NIS Elements AR Analysis 5.20.02 software and automated using the JOBS module. In total, 500–800 cells were evaluated. An example of image segmentation is shown in Figure S2 (Supplementary Materials).

#### 4.3.4. Evaluation of Young’s Modulus as Indicator for MP Uptake

In the comparison of the Young’s moduli from all cancer cells or cancer cell spheroids, the different numbers of force curves generated for the various cell lines and culture and exposure conditions were taken into account. To ensure that the MP-exposed and non-exposed groups had a similar distribution of cell lines, Excel’s random number generator was used to select the 100 values included in the comparisons.

### 4.4. Statistics

The Young’s moduli of the spheroids were pooled from different experiments. Since significant variation was noted between the experiments, exposures were repeated for several spheroids. However, irrespective of the number of experiments (3–12), variation was higher in some cell lines, and there was no correlation between variation and the number of experiments (see Figure S3 in the Supplementary Materials). The inter-experiment variation for all spheroids was 19.8 ± 7.4%. For the monolayer experiments, data from only three to four experiments were pooled. In the monolayer experiments, the means of Young’s modulus varied by 23.7 ± 10.6% between different experiments. The data were analyzed using SPSS 29.0.0 software (IBM Austria, Vienna, Austria). As indicated by the Shapiro–Wilk test, the data did not follow a normal distribution. Therefore, data were analyzed using the Kruskal–Wallis test and are shown as box plots. Groups with means that differed with *p*-values < 0.05 were considered to be statistically significant. Correlations between the different parameters (e.g., Young’s moduli of spheroids with different composition, MP uptake, exposure condition) were analyzed using the Spearman correlation coefficient for non-normally distributed data.

## 5. Conclusions

The inclusion of mechanical data, such as cell stiffness, proved a valuable predictor of MP uptake: cells with lower Young’s moduli ingested more MPs. The differences between static and dynamic exposures may reveal variations in plasma membrane composition that are linked to the attachment of plastic particles. Young’s modulus identified MP uptake in some cell lines in monolayer culture and was therefore able to discriminate between these cells. However, more experiments using dynamic exposure to different concentrations of fluorescent MPs are needed to conclude that detection by Young’s modulus is more sensitive than fluorescent analysis. Furthermore, the data indicated that MP uptake could be associated with a more malignant phenotype. The method also appears suitable to demonstrate that spheroids took up MPs. However, the method is technically challenging, has low throughput, and the signal-to-noise ratio is insufficient for quantification. Using transfected cells, future experiments could identify which cells in the spheroids contribute most to the increase in Young’s modulus.

## Data Availability

All analytical results generated during this study are presented in the manuscript. The raw data supporting the conclusions of this article will be made available by the authors on request.

## Supplementary Materials

The supporting information can be downloaded at https://www.mdpi.com/article/10.3390/ijms27093770/s1.

## Abbreviations

The following abbreviations are used in this manuscript:

AFM	Atomic force microscopy
DANS	4-dimethylamino-4′-nitrostilbene
DMEM	Dulbecco’s Modified Eagle Medium
IQR	Interquartile range
MAD	Median absolute deviation
MIPs	Maximum intensity projection
MP	Microplastic particle
