# Supplementary material for: Force Spectroscopy by Atomic Force Microscopy as Indicator for Cellular Microplastic Uptake
Source: Int J Mol Sci. 2026 Apr 23;27(9):3770. doi: 10.3390/ijms27093770 (PMC13164443; doi:10.3390/ijms27093770)
Supplement: Supplementary file 1 [file ijms-27-03770-s001.zip › ijms-4238481-supplementary.pdf]

Force Spectroscopy by Atomic Force Microscopy as Indicator for Cellular Microplastic Uptake

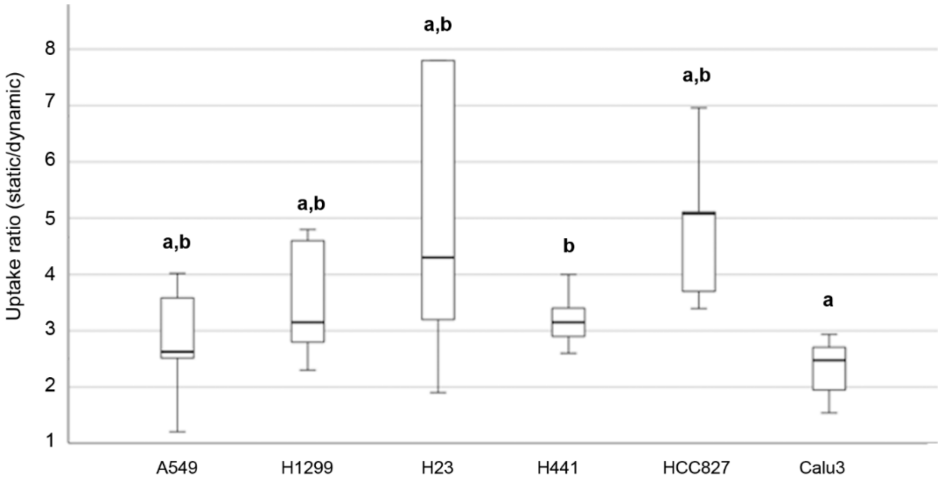

Figure S1: Ratio of ingested MPs in static and dynamic exposure in cell monolayers. Groups with significant differences ($p < 0.05$) are designated by letters.

Cryostat and paraffin sections were used to characterize the composition of the spheroids. The paraffin sections required antigen retrieval after paraffin removal. This was performed in IHC Target Retrieval Solution, pH 6 (Thermo Fisher Scientific, Vienna, Austria), at 110 °C for 10-15 min in a decloaking chamber (Biocare Medical, Pacheco, USA). The staining was similar for cryostat and paraffin sections. Permeabilization was performed with 0.1% Triton X-100 in PBS for 20 min at RT. After rinsing with PBS, the primary antibodies were added. Mouse anti-fibronectin antibody (Sigma-Aldrich, Vienna, Austria, 1:500) was added for 18h at 4°C, rabbit anti-cytokeratin 18 antibody (Sigma-Aldrich, Vienna, Austria, 1:500) for 90 min at RT, and PE anti-CD45 antibody (BD Biosciences, Vienna, Austria, 1:500) for 60 min at RT. Mouse IgG (Linaris, Dossenheim, Germany,1:1000) and rabbit IgG (Linaris, 1:1000) served as negative controls. After each antibody, sections were rinsed three times with PBS. The secondary antibodies goat anti-mouse Alexa Fluor 488 (Thermo Fisher Scientific, 1:500) and goat anti-rabbit Alexa Fluor 635 (Thermo Fisher Scientific, 1:500) were incubated for 30 min at RT. Hoechst 33342 (1 µg/mL) was added to the second antibody solution for nuclear staining. Slides were mounted in fluorescence-mounting medium (Dako, Hamburg, Germany). Images were taken at Nikon A1R confocal microscope with Plan Apo λ 20x objective and acquired at ex/em of 409 nm/450nm for Hoechst 33342 and 488 nm/525 nm for Alexa Fluor 488, 562 nm/595 nm for Alexa Fluor 635 and 641 nm/700 nm for PE.

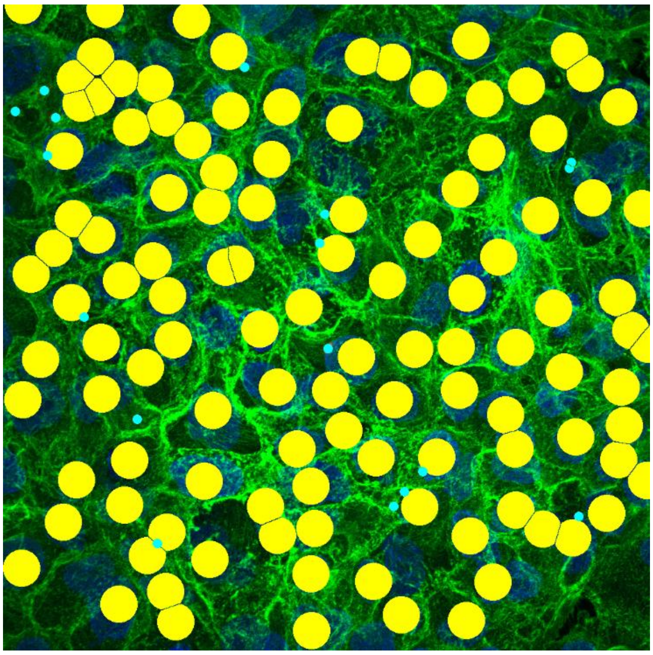

Figure S2: Image segmentation showing identification of nuclei (yellow) and particles (turquoise) in the maximum intensity projection of one z-stack (10 µm thickness) in one region of interest. Nikon image analysis software was used for segmentation and object counting and JOBS module was used to increase throughput.

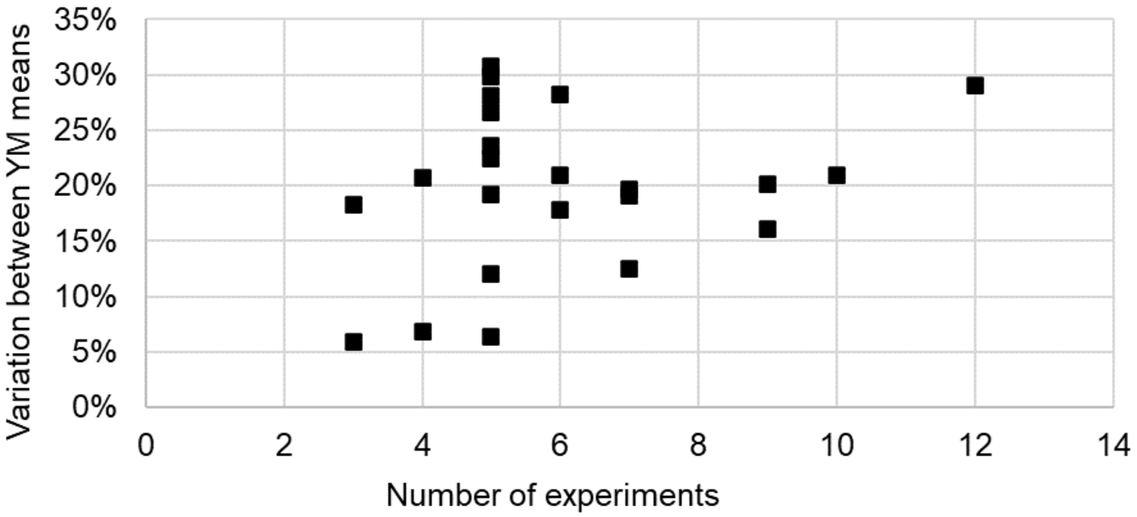

Figure S3: Variation of the means in Young's modulus (YM) obtained from different numbers of experiments. There is no obvious negative correlation between variation and the number of experiments.
